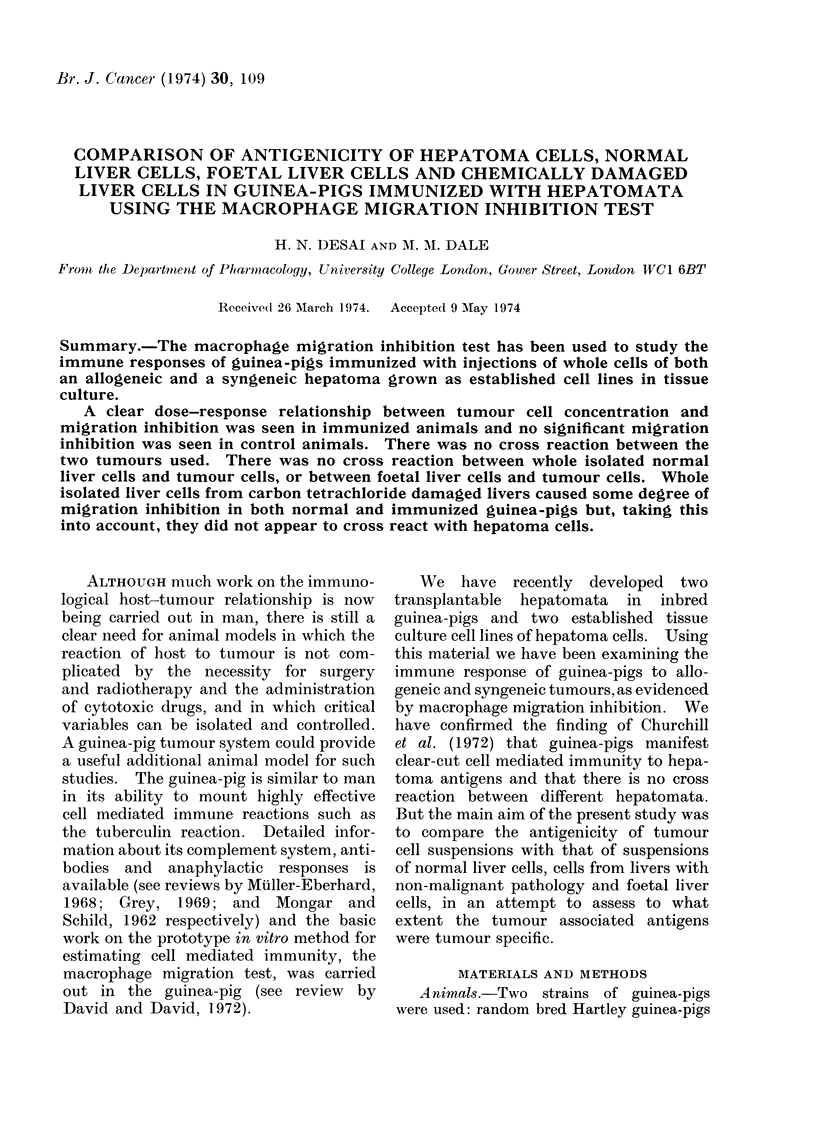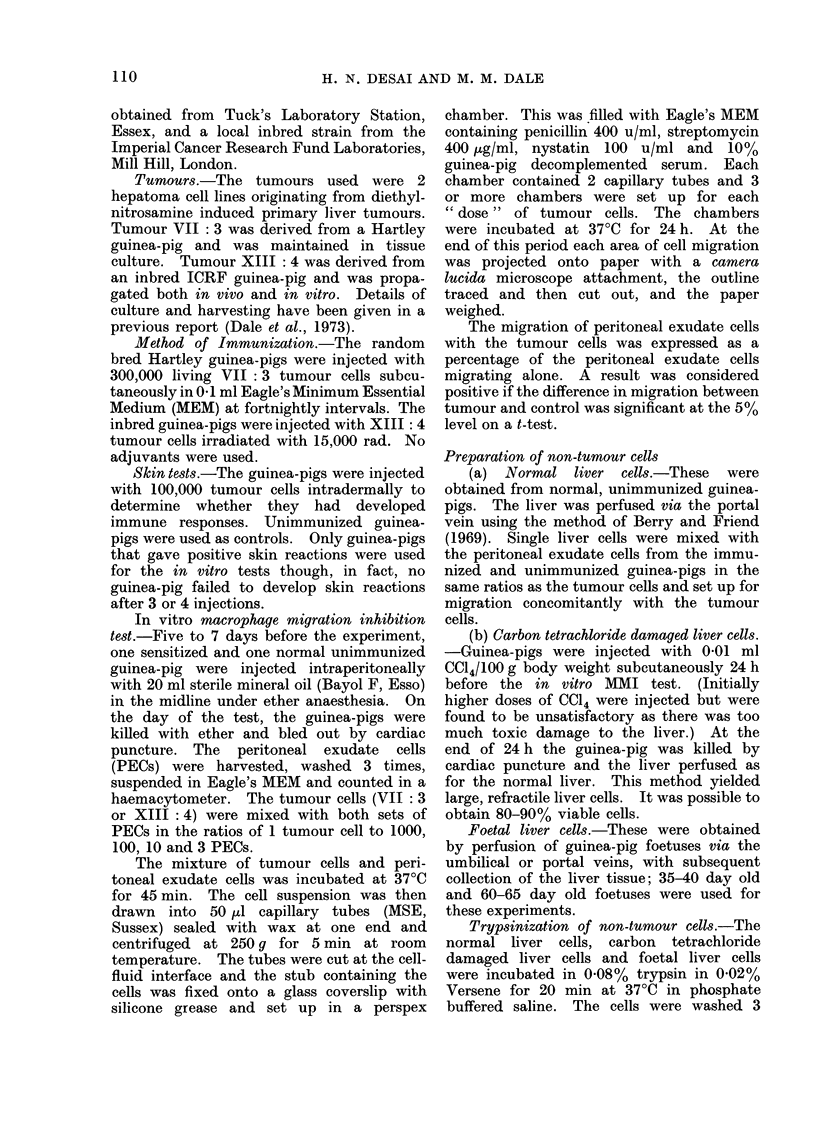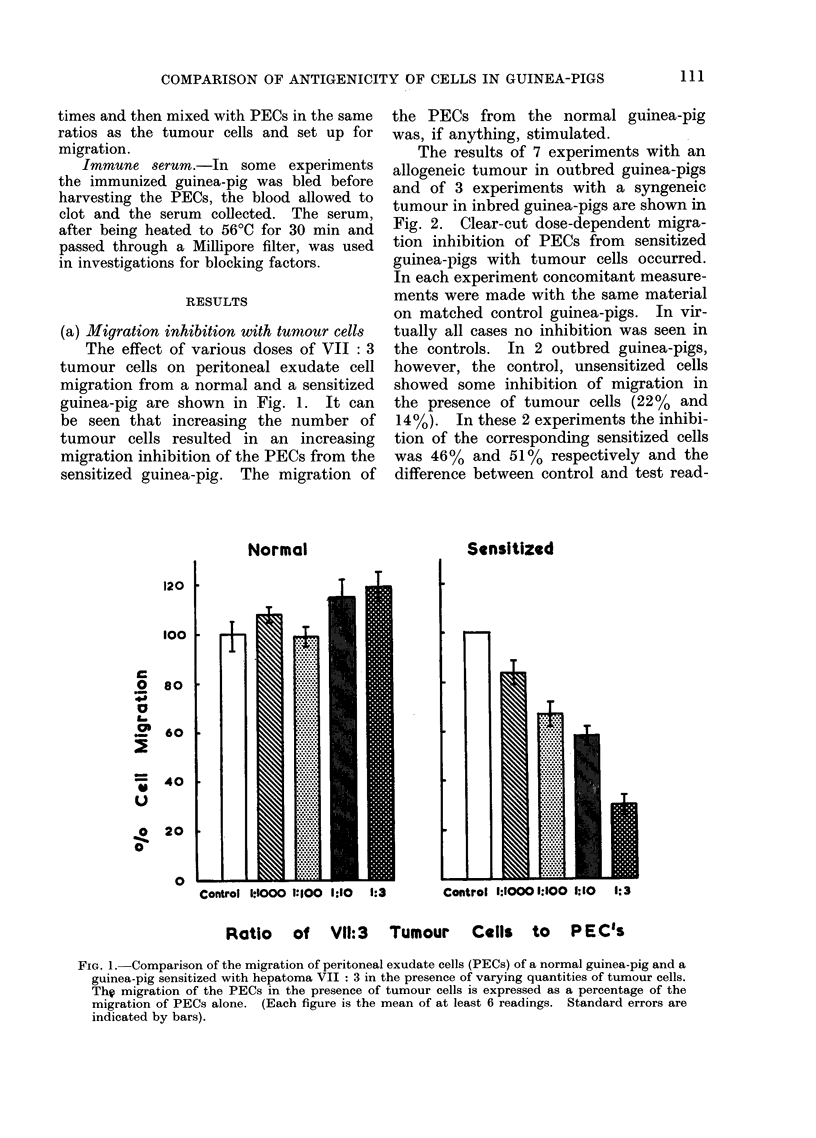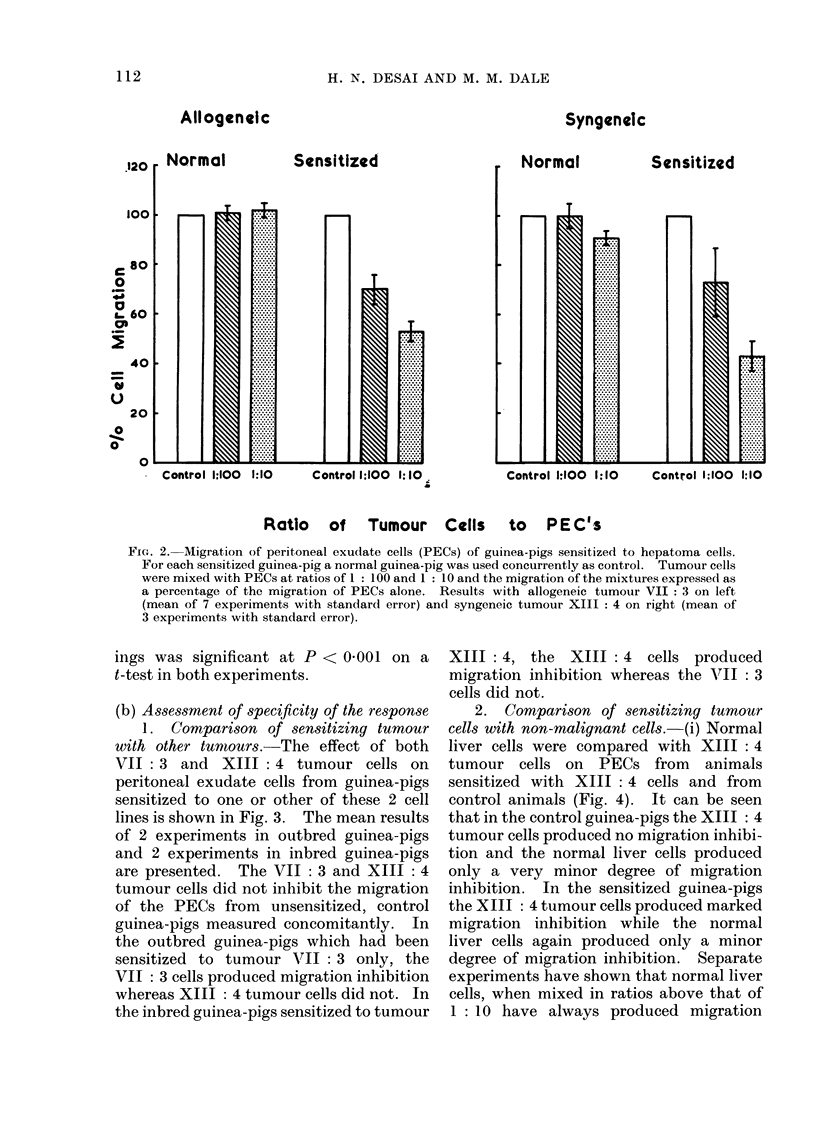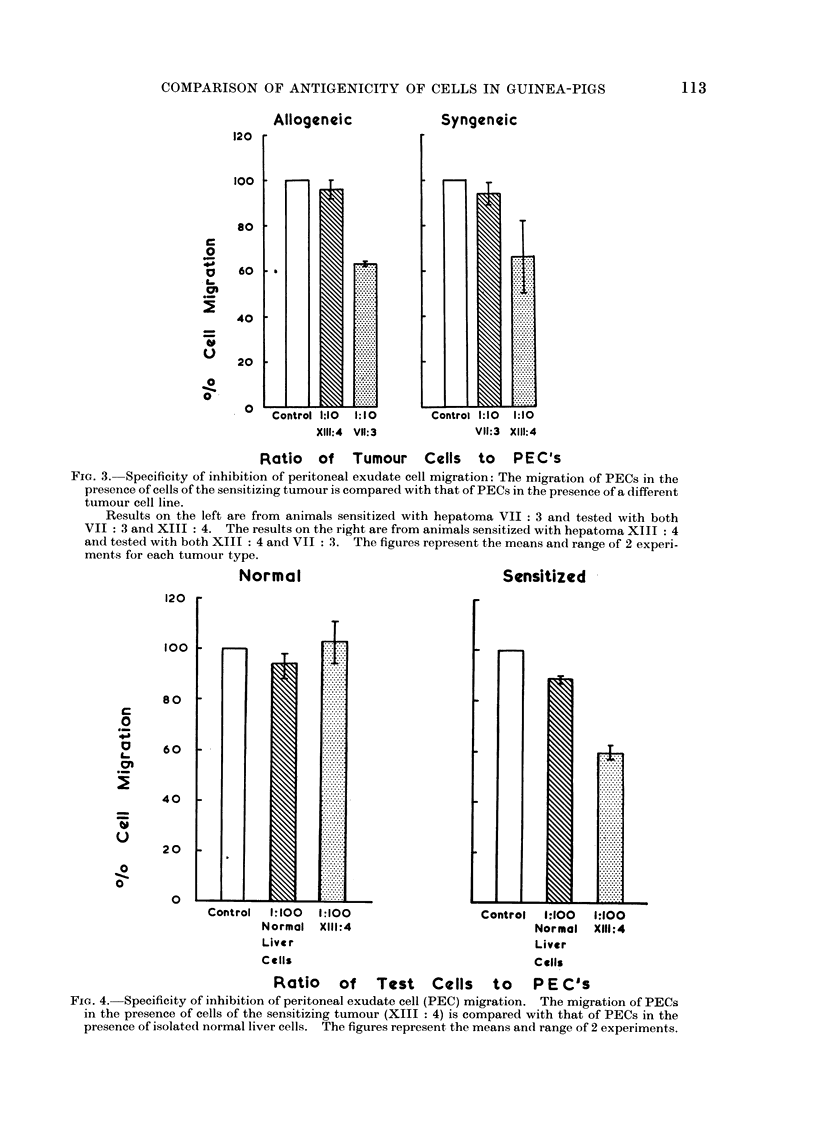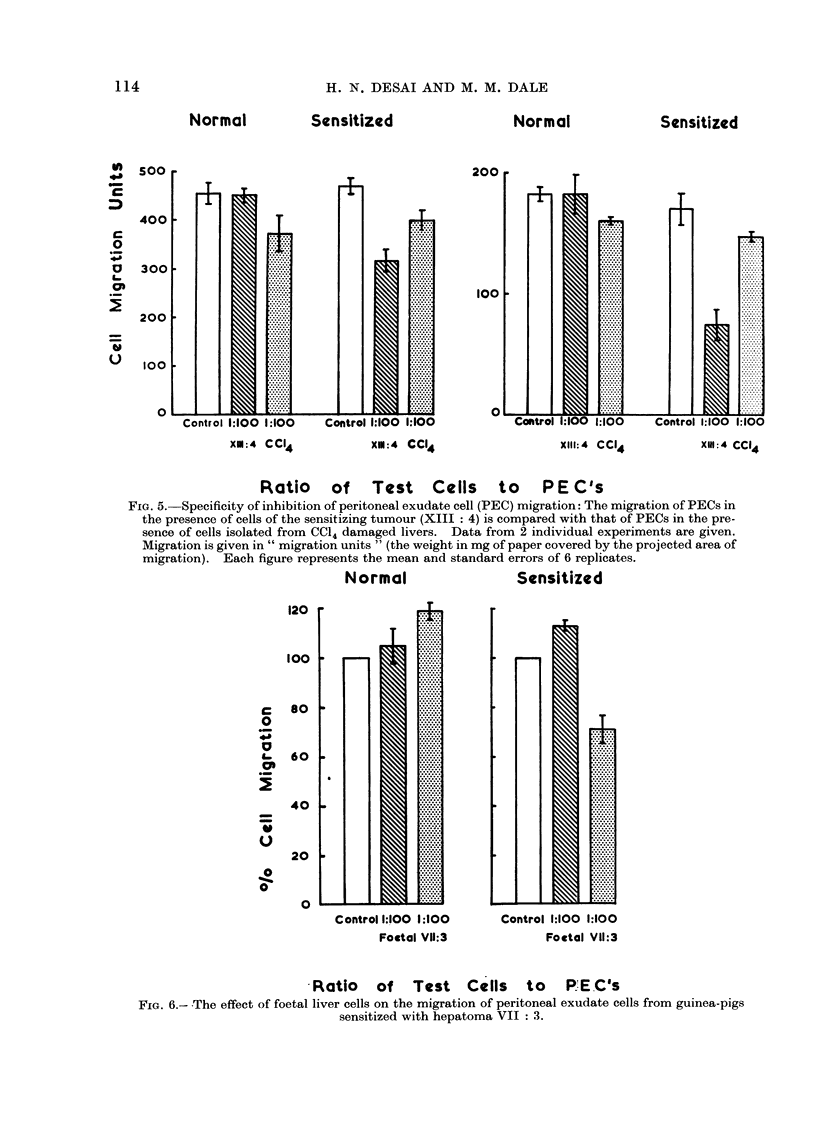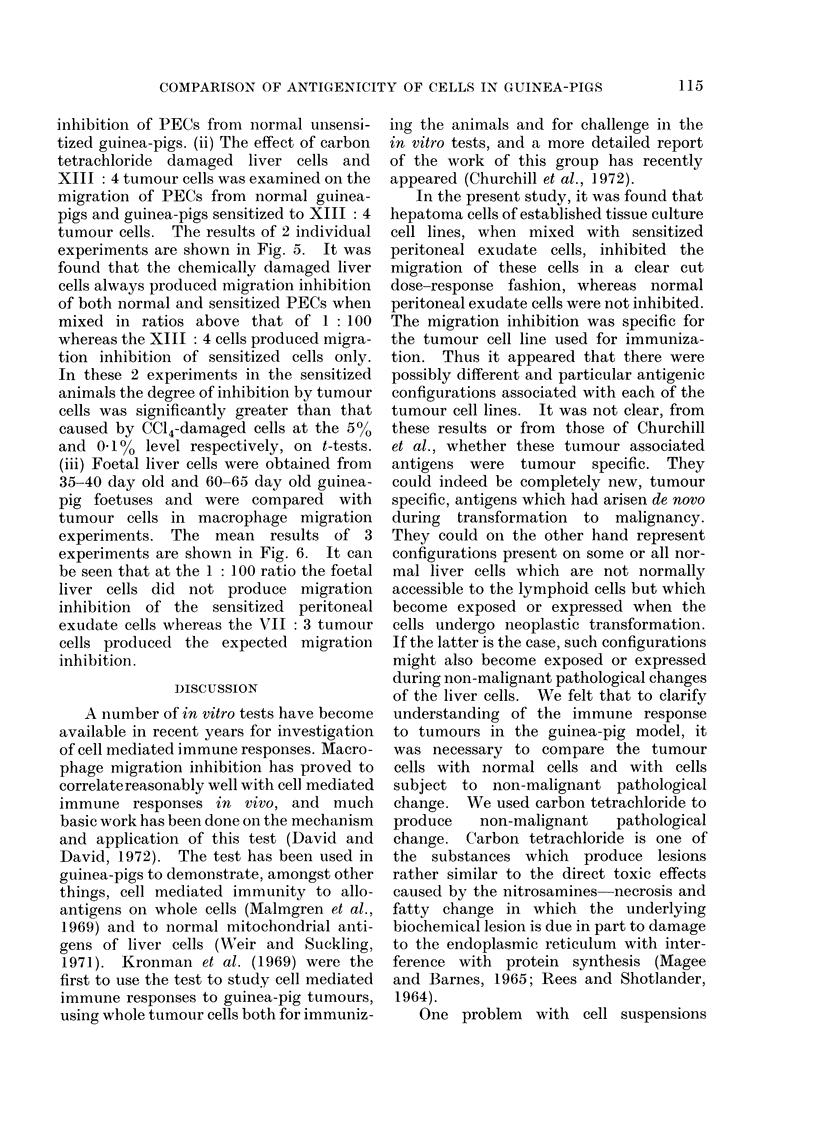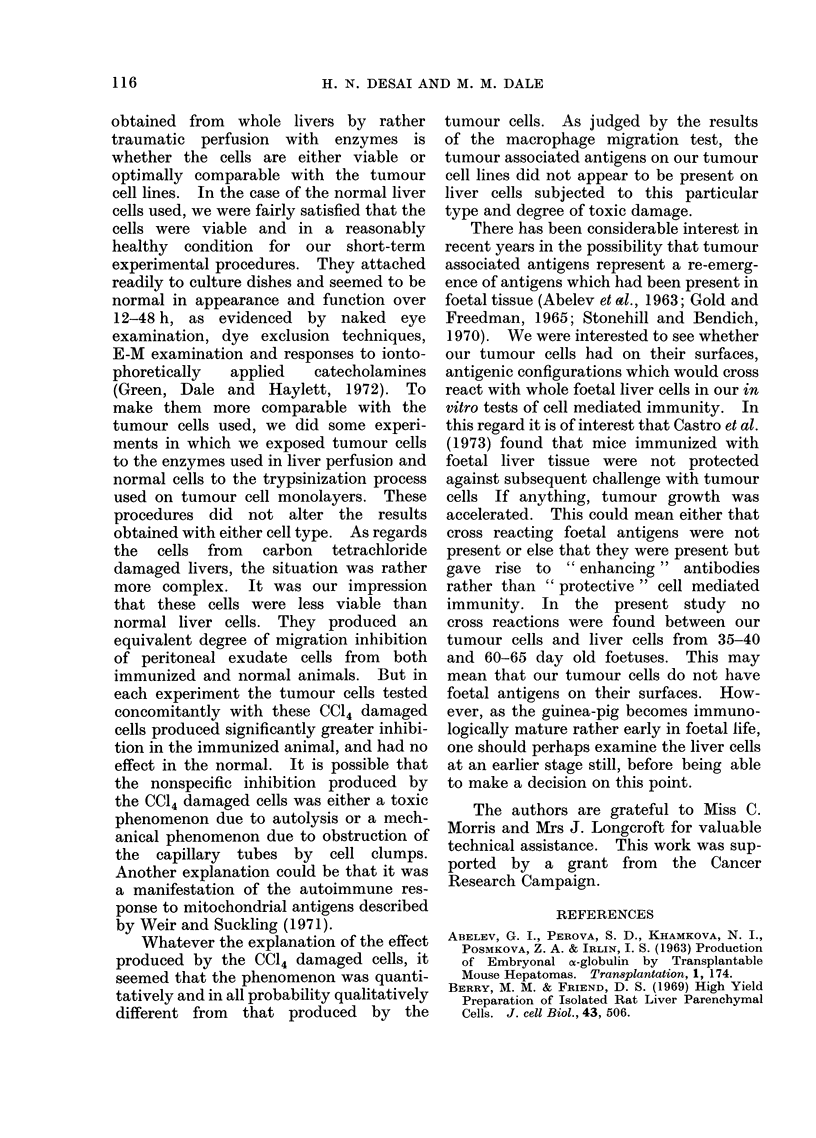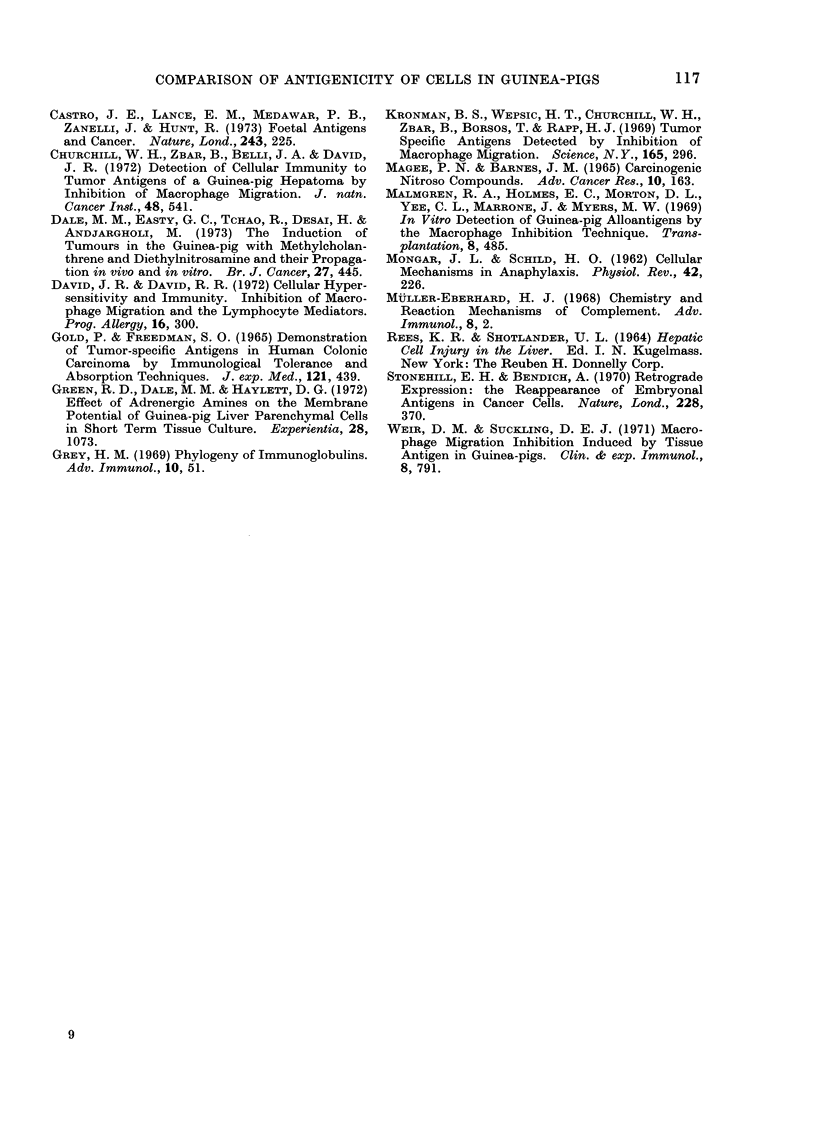# Comparison of Antigenicity of Hepatoma Cells, Normal Liver Cells, Foetal Liver Cells and Chemically Damaged Liver Cells in Guinea-pigs Immunized with Hepatomata using the Macrophage Migration Inhibition Test

**DOI:** 10.1038/bjc.1974.121

**Published:** 1974-08

**Authors:** H. N. Desai, M. M. Dale

## Abstract

The macrophage migration inhibition test has been used to study the immune responses of guinea-pigs immunized with injections of whole cells of both an allogeneic and a syngeneic hepatoma grown as established cell lines in tissue culture.

A clear dose-response relationship between tumour cell concentration and migration inhibition was seen in immunized animals and no significant migration inhibition was seen in control animals. There was no cross reaction between the two tumours used. There was no cross reaction between whole isolated normal liver cells and tumour cells, or between foetal liver cells and tumour cells. Whole isolated liver cells from carbon tetrachloride damaged livers caused some degree of migration inhibition in both normal and immunized guinea-pigs but, taking this into account, they did not appear to cross react with hepatoma cells.


*Br. J. Cancer* (1974) **30**, 109

### COMPARISON OF ANTIGENICITY OF HEPATOMA CELLS, NORMAL LIVER CELLS, FOETAL LIVER CELLS AND CHEMICALLY DAMAGED LIVER CELLS IN GUINEA-PIGS IMMUNIZED WITH HEPATOMATA USING THE MACROPHAGE MIGRATION INHIBITION TEST

H. N. DESAI AND M. M. DALE

*From the Department of Pharmacology, University College London, Gower Street, London WC1 6BT*

Received 26 March 1974. Accepted 9 May 1974

**Summary.—The macrophage migration inhibition test has been used to study the immune responses of guinea-pigs immunized with injections of whole cells of both an allogeneic and a syngeneic hepatoma grown as established cell lines in tissue culture.**

**A clear dose–response relationship between tumour cell concentration and migration inhibition was seen in immunized animals and no significant migration inhibition was seen in control animals. There was no cross reaction between the two tumours used. There was no cross reaction between whole isolated normal liver cells and tumour cells, or between foetal liver cells and tumour cells. Whole isolated liver cells from carbon tetrachloride damaged livers caused some degree of migration inhibition in both normal and immunized guinea-pigs but, taking this into account, they did not appear to cross react with hepatoma cells.**

ALTHOUGH much work on the immunological host–tumour relationship is now being carried out in man, there is still a clear need for animal models in which the reaction of host to tumour is not complicated by the necessity for surgery and radiotherapy and the administration of cytotoxic drugs, and in which critical variables can be isolated and controlled. A guinea-pig tumour system could provide a useful additional animal model for such studies. The guinea-pig is similar to man in its ability to mount highly effective cell mediated immune reactions such as the tuberculin reaction. Detailed information about its complement system, antibodies and anaphylactic responses is available (see reviews by Müller-Eberhard, 1968; Grey, 1969; and Mongar and Schild, 1962 respectively) and the basic work on the prototype *in vitro* method for estimating cell mediated immunity, the macrophage migration test, was carried out in the guinea-pig (see review by David and David, 1972).

We have recently developed two transplantable hepatomata in inbred guinea-pigs and two established tissue culture cell lines of hepatoma cells. Using this material we have been examining the immune response of guinea-pigs to allogeneic and syngeneic tumours, as evidenced by macrophage migration inhibition. We have confirmed the finding of Churchill *et al.* (1972) that guinea-pigs manifest clear-cut cell mediated immunity to hepatoma antigens and that there is no cross reaction between different hepatomata. But the main aim of the present study was to compare the antigenicity of tumour cell suspensions with that of suspensions of normal liver cells, cells from livers with non-malignant pathology and foetal liver cells, in an attempt to assess to what extent the tumour associated antigens were tumour specific.

### MATERIALS AND METHODS

*Animals.*—Two strains of guinea-pigs were used: random bred Hartley guinea-pigs obtained from Tuck's Laboratory Station, Essex, and a local inbred strain from the Imperial Cancer Research Fund Laboratories, Mill Hill, London.

*Tumours.*—The tumours used were 2 hepatoma cell lines originating from diethylnitrosamine induced primary liver tumours. Tumour VII : 3 was derived from a Hartley guinea-pig and was maintained in tissue culture. Tumour XIII : 4 was derived from an inbred ICRF guinea-pig and was propagated both *in vivo* and *in vitro*. Details of culture and harvesting have been given in a previous report (Dale *et al.*, 1973).

*Method of Immunization.*—The random bred Hartley guinea-pigs were injected with 300,000 living VII : 3 tumour cells subcutaneously in 0·1 ml Eagle's Minimum Essential Medium (MEM) at fortnightly intervals. The inbred guinea-pigs were injected with XIII : 4 tumour cells irradiated with 15,000 rad. No adjuvants were used.

*Skin tests.*—The guinea-pigs were injected with 100,000 tumour cells intradermally to determine whether they had developed immune responses. Unimmunized guinea-pigs were used as controls. Only guinea-pigs that gave positive skin reactions were used for the *in vitro* tests though, in fact, no guinea-pig failed to develop skin reactions after 3 or 4 injections.

In vitro *macrophage migration inhibition test.*—Five to 7 days before the experiment, one sensitized and one normal unimmunized guinea-pig were injected intraperitoneally with 20 ml sterile mineral oil (Bayol F, Esso) in the midline under ether anaesthesia. On the day of the test, the guinea-pigs were killed with ether and bled out by cardiac puncture. The peritoneal exudate cells (PECs) were harvested, washed 3 times, suspended in Eagle's MEM and counted in a haemacytometer. The tumour cells (VII : 3 or XIII : 4) were mixed with both sets of PECs in the ratios of 1 tumour cell to 1000, 100, 10 and 3 PECs.

The mixture of tumour cells and peritoneal exudate cells was incubated at 37°C for 45 min. The cell suspension was then drawn into 50 $\mu$l capillary tubes (MSE, Sussex) sealed with wax at one end and centrifuged at 250 *g* for 5 min at room temperature. The tubes were cut at the cell-fluid interface and the stub containing the cells was fixed onto a glass coverslip with silicone grease and set up in a perspex chamber. This was filled with Eagle's MEM containing penicillin 400 u/ml, streptomycin 400 $\mu$g/ml, nystatin 100 u/ml and 10% guinea-pig decomplemented serum. Each chamber contained 2 capillary tubes and 3 or more chambers were set up for each "dose" of tumour cells. The chambers were incubated at 37°C for 24 h. At the end of this period each area of cell migration was projected onto paper with a *camera lucida* microscope attachment, the outline traced and then cut out, and the paper weighed.

The migration of peritoneal exudate cells with the tumour cells was expressed as a percentage of the peritoneal exudate cells migrating alone. A result was considered positive if the difference in migration between tumour and control was significant at the 5% level on a *t*-test.

*Preparation of non-tumour cells*

(a) *Normal liver cells.*—These were obtained from normal, unimmunized guinea-pigs. The liver was perfused *via* the portal vein using the method of Berry and Friend (1969). Single liver cells were mixed with the peritoneal exudate cells from the immunized and unimmunized guinea-pigs in the same ratios as the tumour cells and set up for migration concomitantly with the tumour cells.

(b) *Carbon tetrachloride damaged liver cells.*—Guinea-pigs were injected with 0·01 ml $CCl_4$/100 g body weight subcutaneously 24 h before the *in vitro* MMI test. (Initially higher doses of $CCl_4$ were injected but were found to be unsatisfactory as there was too much toxic damage to the liver.) At the end of 24 h the guinea-pig was killed by cardiac puncture and the liver perfused as for the normal liver. This method yielded large, refractile liver cells. It was possible to obtain 80–90% viable cells.

*Foetal liver cells.*—These were obtained by perfusion of guinea-pig foetuses *via* the umbilical or portal veins, with subsequent collection of the liver tissue; 35–40 day old and 60–65 day old foetuses were used for these experiments.

*Trypsinization of non-tumour cells.*—The normal liver cells, carbon tetrachloride damaged liver cells and foetal liver cells were incubated in 0·08% trypsin in 0·02% Versene for 20 min at 37°C in phosphate buffered saline. The cells were washed 3

times and then mixed with PECs in the same ratios as the tumour cells and set up for migration.

*Immune serum.*—In some experiments the immunized guinea-pig was bled before harvesting the PECs, the blood allowed to clot and the serum collected. The serum, after being heated to 56°C for 30 min and passed through a Millipore filter, was used in investigations for blocking factors.

### RESULTS

### (a) *Migration inhibition with tumour cells*

The effect of various doses of VII : 3 tumour cells on peritoneal exudate cell migration from a normal and a sensitized guinea-pig are shown in Fig. 1. It can be seen that increasing the number of tumour cells resulted in an increasing migration inhibition of the PECs from the sensitized guinea-pig. The migration of the PECs from the normal guinea-pig was, if anything, stimulated.

The results of 7 experiments with an allogeneic tumour in outbred guinea-pigs and of 3 experiments with a syngeneic tumour in inbred guinea-pigs are shown in Fig. 2. Clear-cut dose-dependent migration inhibition of PECs from sensitized guinea-pigs with tumour cells occurred. In each experiment concomitant measurements were made with the same material on matched control guinea-pigs. In virtually all cases no inhibition was seen in the controls. In 2 outbred guinea-pigs, however, the control, unsensitized cells showed some inhibition of migration in the presence of tumour cells (22% and 14%). In these 2 experiments the inhibition of the corresponding sensitized cells was 46% and 51% respectively and the difference between control and test read-

FIG. 1.—Comparison of the migration of peritoneal exudate cells (PECs) of a normal guinea-pig and a guinea-pig sensitized with hepatoma VII : 3 in the presence of varying quantities of tumour cells. The migration of the PECs in the presence of tumour cells is expressed as a percentage of the migration of PECs alone. (Each figure is the mean of at least 6 readings. Standard errors are indicated by bars).

FIG. 2.—Migration of peritoneal exudate cells (PECs) of guinea-pigs sensitized to hepatoma cells. For each sensitized guinea-pig a normal guinea-pig was used concurrently as control. Tumour cells were mixed with PECs at ratios of 1 : 100 and 1 : 10 and the migration of the mixtures expressed as a percentage of the migration of PECs alone. Results with allogeneic tumour VII : 3 on left (mean of 7 experiments with standard error) and syngeneic tumour XIII : 4 on right (mean of 3 experiments with standard error).

ings was significant at $P < 0{\cdot}001$ on a $t$-test in both experiments.

(b) *Assessment of specificity of the response*

1. *Comparison of sensitizing tumour with other tumours.*—The effect of both VII : 3 and XIII : 4 tumour cells on peritoneal exudate cells from guinea-pigs sensitized to one or other of these 2 cell lines is shown in Fig. 3. The mean results of 2 experiments in outbred guinea-pigs and 2 experiments in inbred guinea-pigs are presented. The VII : 3 and XIII : 4 tumour cells did not inhibit the migration of the PECs from unsensitized, control guinea-pigs measured concomitantly. In the outbred guinea-pigs which had been sensitized to tumour VII : 3 only, the VII : 3 cells produced migration inhibition whereas XIII : 4 tumour cells did not. In the inbred guinea-pigs sensitized to tumour XIII : 4, the XIII : 4 cells produced migration inhibition whereas the VII : 3 cells did not.

2. *Comparison of sensitizing tumour cells with non-malignant cells.*—(i) Normal liver cells were compared with XIII : 4 tumour cells on PECs from animals sensitized with XIII : 4 cells and from control animals (Fig. 4). It can be seen that in the control guinea-pigs the XIII : 4 tumour cells produced no migration inhibition and the normal liver cells produced only a very minor degree of migration inhibition. In the sensitized guinea-pigs the XIII : 4 tumour cells produced marked migration inhibition while the normal liver cells again produced only a minor degree of migration inhibition. Separate experiments have shown that normal liver cells, when mixed in ratios above that of 1 : 10 have always produced migration

FIG. 3.—Specificity of inhibition of peritoneal exudate cell migration: The migration of PECs in the presence of cells of the sensitizing tumour is compared with that of PECs in the presence of a different tumour cell line.

Results on the left are from animals sensitized with hepatoma VII : 3 and tested with both VII : 3 and XIII : 4. The results on the right are from animals sensitized with hepatoma XIII : 4 and tested with both XIII : 4 and VII : 3. The figures represent the means and range of 2 experiments for each tumour type.

FIG. 4.—Specificity of inhibition of peritoneal exudate cell (PEC) migration. The migration of PECs in the presence of cells of the sensitizing tumour (XIII : 4) is compared with that of PECs in the presence of isolated normal liver cells. The figures represent the means and range of 2 experiments.

FIG. 5.—Specificity of inhibition of peritoneal exudate cell (PEC) migration: The migration of PECs in the presence of cells of the sensitizing tumour (XIII : 4) is compared with that of PECs in the presence of cells isolated from $CCl_4$ damaged livers. Data from 2 individual experiments are given. Migration is given in " migration units " (the weight in mg of paper covered by the projected area of migration). Each figure represents the mean and standard errors of 6 replicates.

FIG. 6.—The effect of foetal liver cells on the migration of peritoneal exudate cells from guinea-pigs sensitized with hepatoma VII : 3.

inhibition of PECs from normal unsensitized guinea-pigs. (ii) The effect of carbon tetrachloride damaged liver cells and XIII : 4 tumour cells was examined on the migration of PECs from normal guinea-pigs and guinea-pigs sensitized to XIII : 4 tumour cells. The results of 2 individual experiments are shown in Fig. 5. It was found that the chemically damaged liver cells always produced migration inhibition of both normal and sensitized PECs when mixed in ratios above that of 1 : 100 whereas the XIII : 4 cells produced migration inhibition of sensitized cells only. In these 2 experiments in the sensitized animals the degree of inhibition by tumour cells was significantly greater than that caused by $CCl_4$-damaged cells at the 5% and 0·1% level respectively, on *t*-tests. (iii) Foetal liver cells were obtained from 35–40 day old and 60–65 day old guinea-pig foetuses and were compared with tumour cells in macrophage migration experiments. The mean results of 3 experiments are shown in Fig. 6. It can be seen that at the 1 : 100 ratio the foetal liver cells did not produce migration inhibition of the sensitized peritoneal exudate cells whereas the VII : 3 tumour cells produced the expected migration inhibition.

### DISCUSSION

A number of *in vitro* tests have become available in recent years for investigation of cell mediated immune responses. Macrophage migration inhibition has proved to correlate reasonably well with cell mediated immune responses *in vivo*, and much basic work has been done on the mechanism and application of this test (David and David, 1972). The test has been used in guinea-pigs to demonstrate, amongst other things, cell mediated immunity to allo-antigens on whole cells (Malmgren *et al.*, 1969) and to normal mitochondrial antigens of liver cells (Weir and Suckling, 1971). Kronman *et al.* (1969) were the first to use the test to study cell mediated immune responses to guinea-pig tumours, using whole tumour cells both for immunizing the animals and for challenge in the *in vitro* tests, and a more detailed report of the work of this group has recently appeared (Churchill *et al.*, 1972).

In the present study, it was found that hepatoma cells of established tissue culture cell lines, when mixed with sensitized peritoneal exudate cells, inhibited the migration of these cells in a clear cut dose–response fashion, whereas normal peritoneal exudate cells were not inhibited. The migration inhibition was specific for the tumour cell line used for immunization. Thus it appeared that there were possibly different and particular antigenic configurations associated with each of the tumour cell lines. It was not clear, from these results or from those of Churchill *et al.*, whether these tumour associated antigens were tumour specific. They could indeed be completely new, tumour specific, antigens which had arisen *de novo* during transformation to malignancy. They could on the other hand represent configurations present on some or all normal liver cells which are not normally accessible to the lymphoid cells but which become exposed or expressed when the cells undergo neoplastic transformation. If the latter is the case, such configurations might also become exposed or expressed during non-malignant pathological changes of the liver cells. We felt that to clarify understanding of the immune response to tumours in the guinea-pig model, it was necessary to compare the tumour cells with normal cells and with cells subject to non-malignant pathological change. We used carbon tetrachloride to produce non-malignant pathological change. Carbon tetrachloride is one of the substances which produce lesions rather similar to the direct toxic effects caused by the nitrosamines—necrosis and fatty change in which the underlying biochemical lesion is due in part to damage to the endoplasmic reticulum with interference with protein synthesis (Magee and Barnes, 1965; Rees and Shotlander, 1964).

One problem with cell suspensions obtained from whole livers by rather traumatic perfusion with enzymes is whether the cells are either viable or optimally comparable with the tumour cell lines. In the case of the normal liver cells used, we were fairly satisfied that the cells were viable and in a reasonably healthy condition for our short-term experimental procedures. They attached readily to culture dishes and seemed to be normal in appearance and function over 12–48 h, as evidenced by naked eye examination, dye exclusion techniques, E-M examination and responses to iontophoretically applied catecholamines (Green, Dale and Haylett, 1972). To make them more comparable with the tumour cells used, we did some experiments in which we exposed tumour cells to the enzymes used in liver perfusion and normal cells to the trypsinization process used on tumour cell monolayers. These procedures did not alter the results obtained with either cell type. As regards the cells from carbon tetrachloride damaged livers, the situation was rather more complex. It was our impression that these cells were less viable than normal liver cells. They produced an equivalent degree of migration inhibition of peritoneal exudate cells from both immunized and normal animals. But in each experiment the tumour cells tested concomitantly with these $CCl_4$ damaged cells produced significantly greater inhibition in the immunized animal, and had no effect in the normal. It is possible that the nonspecific inhibition produced by the $CCl_4$ damaged cells was either a toxic phenomenon due to autolysis or a mechanical phenomenon due to obstruction of the capillary tubes by cell clumps. Another explanation could be that it was a manifestation of the autoimmune response to mitochondrial antigens described by Weir and Suckling (1971).

Whatever the explanation of the effect produced by the $CCl_4$ damaged cells, it seemed that the phenomenon was quantitatively and in all probability qualitatively different from that produced by the tumour cells. As judged by the results of the macrophage migration test, the tumour associated antigens on our tumour cell lines did not appear to be present on liver cells subjected to this particular type and degree of toxic damage.

There has been considerable interest in recent years in the possibility that tumour associated antigens represent a re-emergence of antigens which had been present in foetal tissue (Abelev *et al.*, 1963; Gold and Freedman, 1965; Stonehill and Bendich, 1970). We were interested to see whether our tumour cells had on their surfaces, antigenic configurations which would cross react with whole foetal liver cells in our *in vitro* tests of cell mediated immunity. In this regard it is of interest that Castro *et al.* (1973) found that mice immunized with foetal liver tissue were not protected against subsequent challenge with tumour cells If anything, tumour growth was accelerated. This could mean either that cross reacting foetal antigens were not present or else that they were present but gave rise to " enhancing " antibodies rather than " protective " cell mediated immunity. In the present study no cross reactions were found between our tumour cells and liver cells from 35–40 and 60–65 day old foetuses. This may mean that our tumour cells do not have foetal antigens on their surfaces. However, as the guinea-pig becomes immunologically mature rather early in foetal life, one should perhaps examine the liver cells at an earlier stage still, before being able to make a decision on this point.

The authors are grateful to Miss C. Morris and Mrs J. Longcroft for valuable technical assistance. This work was supported by a grant from the Cancer Research Campaign.

### REFERENCES

Abelev, G. I., Perova, S. D., Khamkova, N. I., Posmkova, Z. A. & Irlin, I. S. (1963) Production of Embryonal α-globulin by Transplantable Mouse Hepatomas. *Transplantation*, **1**, 174.

Berry, M. M. & Friend, D. S. (1969) High Yield Preparation of Isolated Rat Liver Parenchymal Cells. *J. cell Biol.*, **43**, 506.

Castro, J. E., Lance, E. M., Medawar, P. B., Zanelli, J. & Hunt, R. (1973) Foetal Antigens and Cancer. *Nature, Lond.*, **243**, 225.

Churchill, W. H., Zbar, B., Belli, J. A. & David, J. R. (1972) Detection of Cellular Immunity to Tumor Antigens of a Guinea-pig Hepatoma by Inhibition of Macrophage Migration. *J. natn. Cancer Inst.*, **48**, 541.

Dale, M. M., Easty, G. C., Tchao, R., Desai, H. & Andjargholi, M. (1973) The Induction of Tumours in the Guinea-pig with Methylcholanthrene and Diethylnitrosamine and their Propagation *in vivo* and *in vitro*. *Br. J. Cancer*, **27**, 445.

David, J. R. & David, R. R. (1972) Cellular Hypersensitivity and Immunity. Inhibition of Macrophage Migration and the Lymphocyte Mediators. *Prog. Allergy*, **16**, 300.

Gold, P. & Freedman, S. O. (1965) Demonstration of Tumor-specific Antigens in Human Colonic Carcinoma by Immunological Tolerance and Absorption Techniques. *J. exp. Med.*, **121**, 439.

Green, R. D., Dale, M. M. & Haylett, D. G. (1972) Effect of Adrenergic Amines on the Membrane Potential of Guinea-pig Liver Parenchymal Cells in Short Term Tissue Culture. *Experientia*, **28**, 1073.

Grey, H. M. (1969) Phylogeny of Immunoglobulins. *Adv. Immunol.*, **10**, 51.

Kronman, B. S., Wepsic, H. T., Churchill, W. H., Zbar, B., Borsos, T. & Rapp, H. J. (1969) Tumor Specific Antigens Detected by Inhibition of Macrophage Migration. *Science, N.Y.*, **165**, 296.

Magee, P. N. & Barnes, J. M. (1965) Carcinogenic Nitroso Compounds. *Adv. Cancer Res.*, **10**, 163.

Malmgren, R. A., Holmes, E. C., Morton, D. L., Yee, C. L., Marrone, J. & Myers, M. W. (1969) *In Vitro* Detection of Guinea-pig Alloantigens by the Macrophage Inhibition Technique. *Transplantation*, **8**, 485.

Mongar, J. L. & Schild, H. O. (1962) Cellular Mechanisms in Anaphylaxis. *Physiol. Rev.*, **42**, 226.

Müller-Eberhard, H. J. (1968) Chemistry and Reaction Mechanisms of Complement. *Adv. Immunol.*, **8**, 2.

Rees, K. R. & Shotlander, U. L. (1964) *Hepatic Cell Injury in the Liver*. Ed. I. N. Kugelmass. New York: The Reuben H. Donnelly Corp.

Stonehill, E. H. & Bendich, A. (1970) Retrograde Expression: the Reappearance of Embryonal Antigens in Cancer Cells. *Nature, Lond.*, **228**, 370.

Weir, D. M. & Suckling, D. E. J. (1971) Macrophage Migration Inhibition Induced by Tissue Antigen in Guinea-pigs. *Clin. & exp. Immunol.*, **8**, 791.